# Global transfusion practices in septic patients in the intensive care unit: insights from the InPUT‐study sub‐analysis

**DOI:** 10.1111/trf.18445

**Published:** 2025-10-22

**Authors:** Vincent C. Kurucz, Andrew W. J. Flint, Alexis Poole, Merijn C. Reuland, Claudia van den Oord, Caroline M. Schaap, Jan Bakker, Maurizio Cecconi, Aarne Feldheiser, Jens Meier, Zoe McQuilten, Marcella C. A. Müller, Sanne de Bruin, Thomas W. L. Scheeren, Tarikul Hamid, Cécile Aubron, Michaël Piagnerelli, Tina Tomić Mahečić, Jan Benes, Lene Russell, Hernan Aguirre‐Bermeo, Konstantina Triantafyllopoulou, Vasiliki Chantziara, Mohan Gurjar, Sheila Nainan Myatra, Vincenzo Pota, Muhammed Elhadi, Ryszard Gawda, Mafalda Mourisco, Marcus Lance, Vojislava Neskovic, Matej Podbregar, Juan V. Llau, Manual Quintana‐Diaz, Maria Cronhjort, Carmen A. Pfortmueller, Nihan Yapici, Nathan Nielsen, Akshay Shah, Harm‐Jan de Grooth, Alexander P. J. Vlaar, Jimmy Schenk, Senta Jorinde Raasveld, for the InPUT Study Group

**Affiliations:** ^1^ Department of Intensive Care Amsterdam University Medical Centers Amsterdam The Netherlands; ^2^ Transfusion Research Unit, School of Public Health and Preventive Medicine Monash University Melbourne Australia; ^3^ The Australian and New Zealand Intensive Care Research Centre (ANZIC‐RC), School of Public Health and Preventive Medicine Monash University Melbourne Australia; ^4^ Department of Intensive Care Adults Erasmus MC University Medical Centers Rotterdam The Netherlands; ^5^ Department of Pulmonary and Critical Care New York University New York New York USA; ^6^ Department of Pulmonary and Critical Care Columbia University New York New York USA; ^7^ Department of Intensive Care Pontificia Universidad Católica de Chile Santiago Chile; ^8^ Department of Anesthesiology and Intensive Care IRCCS Humanitas Research Hospital Milan Italy; ^9^ Department of Anaesthesiology, Intensive Care Medicine and Pain Therapy EvangKliniken Essen‐Mitte, Huyssens‐Stiftung/Knappschaft Essen Germany; ^10^ Department of Anesthesiology and Intensive Care Kepler University Clinic, Kepler University Linz Austria; ^11^ Department of Anesthesiology University Medical Center Groningen Groningen The Netherlands; ^12^ Department of Critical Care Asgar Ali Hospital Dhaka Bangladesh; ^13^ Médecine Intensive Réanimation, CHU de Brest Université de Bretagne Occidentale Brest France; ^14^ Department of Intensive Care, CHU Charleroi Marie Curie Université Libre de Brussels Charleroi Belgium; ^15^ Department of Anesthesiology and Intensive Care University Clinical Hospital Center Zagreb Zagreb Croatia; ^16^ Department of Anesthesiology and Intensive Care Medicine University Hospital and Faculty of Medicine in Plzen‐Charles University Plzen Czech Republic; ^17^ Department of Intensive Care Copenhagen University Hospital, Rigshospitalet Copenhagen Copenhagen Denmark; ^18^ Department of Anesthesia and Intensive Care Medicine Copenhagen University Hospital‐Gentofte Hellerup Denmark; ^19^ Department of Clinical Medicine University of Copenhagen Copenhagen Denmark; ^20^ Unidad de Cuidados Intensivos Hospital Vicente Corral Moscoso Cuenca Ecuador; ^21^ Department of Cardiothoracic Surgery European Interbalkan Medical Center Thessaloniki Greece; ^22^ Intensive Care Unit, First Department of Respiratory Medicine National and Kapodistrian University of Athens, Sotiria Chest Hospital Athens Greece; ^23^ Department of Critical Care Medicine Sanjay Gandhi Postgraduate Institute of Medical Sciences Lucknow India; ^24^ Department of Anesthesiology, Critical Care and Pain, Tata Memorial Hospital Homi Bhabha National Institute Mumbai India; ^25^ Department of Child, General and Specialistic Surgery University of Campania, Luigi Vanvitelli Naples Italy; ^26^ Faculty of Medicine University of Tripoli Tripoli Libya; ^27^ Department of Anesthesiology and Intensive Care, Institute of Medical Sciences University of Opole Opole Poland; ^28^ Department of Intensive Care Centro Hospitalar de Entro o Douro e Vouga Santa Maria da Feira Portugal; ^29^ Department of Anesthesiology Aga Khan University Hospital Nairobi Kenya; ^30^ Department of Anesthesia and Intensive Care Military Medical Academy Belgrade Belgrade Serbia; ^31^ Department for Internal Intensive Care, General Hospital Celje, Medical Faculty University of Ljubljana Ljubljana Slovenia; ^32^ Department of Anesthesiology and Post‐surgical Critical Care University Hospital Doctor Peset Valencia Spain; ^33^ Intensive Care Service Hospital Universitario La Paz Madrid Spain; ^34^ Department of Clinical Science and Education Södersjukhuset, Karolinska Institutet Stockholm Sweden; ^35^ Department of Intensive Care, Inselspital Bern University Hospital and University of Bern Bern Switzerland; ^36^ Department of Anesthesiology and Reanimation, Dr Siyami Ersek Thoracic and Cardiovascular Surgery Center University of Health Sciences Istanbul Turkey; ^37^ Division of Pulmonary, Critical Care and Sleep Medicine, and Section of Transfusion Medicine and Therapeutic Pathology University of New Mexico School of Medicine Albuquerque New Mexico USA; ^38^ Nuffield Department of Clinical Neurosciences University of Oxford Oxford UK; ^39^ Department of Intensive Care Utrecht University Medical Centers Utrecht The Netherlands; ^40^ Department of Epidemiology and Data Science, Amsterdam University Medical Centre, Location AMC, Amsterdam Public Health University of Amsterdam Amsterdam The Netherlands; ^41^ Department of Anesthesiology Amsterdam University Medical Centers Amsterdam The Netherlands

## Abstract

**Background:**

Transfusion practices among intensive care unit (ICU) patients with sepsis vary widely. While restrictive hemoglobin thresholds for red blood cell (RBC) transfusion are well studied, the indications and thresholds for platelet and plasma transfusions remain uncertain.

**Methods:**

We performed a sepsis‐specific sub‐analysis of the *International Point Prevalence Study of Intensive Care Unit Transfusion Practices*, a prospective, multicenter, observational study capturing all adult ICU admissions during four pre‐scheduled weeks between March 2019 and October 2022. Patients admitted with sepsis or septic shock, or who developed sepsis during their ICU stay, were included. We recorded demographics, daily laboratory values, and transfusion triggers. Primary endpoints were the proportions of patients receiving RBCs, platelets, or plasma; secondary endpoints were indications, pre‐transfusion thresholds, and blood loss.

**Results:**

Among 3643 screened patients, 799 (22%) fulfilled sepsis criteria; within this subgroup, 317 (40%) received at least one blood component. RBCs were transfused in 269 patients (34%), primarily to address anemia or hemodynamic instability, at a mean pre‐transfusion hemoglobin of 7.5 ± 1.4 g/dL, consistent with restrictive practice. Platelets were given to 78 patients (10%) for prophylaxis or active bleeding at a median count of 26 × 10^9^ cells/L (interquartile range 16–51 × 10^9^ cells/L). Plasma was administered to 108 patients (14%), half for bleeding control and half for non‐bleeding indications.

**Conclusions:**

This largest international snapshot of septic ICU transfusion practices confirms adherence to restrictive RBC thresholds but reveals substantial variability in platelet and plasma use. These findings underscore the need for targeted trials to refine transfusion guidelines in sepsis.

**Abbreviations**
AABB: advancement of blood and biotherapies
APACHE: acute physiology and chronic health evaluation
aPTT: activated partial thromboplastin time
CI: confidence interval
COVID‐19: Coronavirus Disease 2019
DIC: disseminated intravascular coagulation
ECMO: extracorporeal membrane oxygenation
Hb: hemoglobin
ICON: Intensive Care Over Nations
ICU: intensive care unit
INPUT: International Point Prevalence Study of Intensive Care Unit Transfusion Practices
INR: International Normalized Ratio
IQR: interquartile range
MAP: mean arterial pressure
MTP: massive transfusion protocol
PACER: Platelet Administration Prior to CEntral Line inseRtion
PLT: platelet count
PT: prothrombin time
RBC: red blood cell
SD: standard deviation
SIC: sepsis‐induced coagulopathy
SOFA: sequential organ failure assessment
STROBE: strengthening the reporting of observational studies in epidemiology
VHA: viscoelastic hemostatic assay

## 1 INTRODUCTION

The transfusion of red blood cells (RBCs), platelets, and plasma is a common therapeutic intervention in the intensive care unit (ICU).^1^, ^2^, ^3^ Patients with sepsis often exhibit abnormalities in peripheral blood counts, clotting proteins, and antithrombotic factors, which can manifest in conditions such as anemia, thrombocytopenia, leukopenia, disseminated intravascular coagulation (DIC), and functional deficiencies of coagulation factors.^4^, ^5^, ^6^

RBC transfusions are a cornerstone in managing severe anemia, aiming to restore oxygen delivery to tissues and mitigate potentially compromised tissue oxygenation. While some studies highlight benefits such as improved hemodynamics and oxygenation, concerns persist regarding limited clinical impact and potential adverse effects, including heightened infection risk and immune modulation.^7^, ^8^, ^9^ In recent decades, studies focusing on sepsis have highlighted the safety of restrictive transfusion practices, recommending a 7 g/dL hemoglobin (Hb) threshold for critically ill patients with sepsis.^10^, ^11^, ^12^ Nonetheless, patient heterogeneity, including age, physical reserve, hemodynamic status, comorbidities, and concurrent therapies, likely necessitates tailored transfusion strategies.^1^, ^9^, ^13^

Although platelet and plasma transfusions are frequently used to manage thrombocytopenia and coagulopathy in sepsis,^14^, ^15^ robust evidence guiding the optimal administration of platelets, plasma, or other coagulation factors remains limited. As a result, the most recent Surviving Sepsis Campaign offers only confined recommendations on this topic.^9^ Elevated platelet turnover and consumption in septic patients may reduce the efficacy of platelet transfusions, thereby limiting their potential benefits.^16^ Moreover, platelet transfusions in patients with sepsis have been linked to increased mortality, raising concerns about their overall therapeutic value.^17^ The Platelet Administration prior to CEntral line inseRtion (PACER) trial, which included both ICU and hematology patients with a substantial proportion of sepsis cases, demonstrated that withholding prophylactic platelet transfusion in patients with platelet counts of 10 × 10 to 50 × 10^9^ cells/L more than doubled the risk of clinically significant bleeding after central venous catheter placement. This effect appeared more pronounced in hematology patients, while the benefit for ICU patients was less evident.^18^ Moreover, the effectiveness of plasma transfusion in non‐bleeding critically ill patients remains unproven,^15^, ^19^, ^20^ and current guidelines advise against its routine use.^1^

Despite evidence supporting the restrictive use of RBC, platelet, and plasma transfusions, the application of these restrictive guidelines appears to be frequently debated by physicians, and existing research on transfusion practices is often limited, particularly in sepsis. Existing research on transfusion practices is often restricted to specific blood components or confined to single‐country or single‐center studies,^2^, ^21^, ^22^ offering a fragmented view of global practices. Given the variability in sepsis management, particularly regarding transfusion practices,^2^, ^23^ an international overview is essential to better understand current approaches and to identify gaps in current research and clinical applications. While closer alignment with evidence‐based thresholds is expected for RBC transfusions, greater variability is anticipated in platelet and plasma transfusions.

## 2 METHODS

### 2.1 Study design and population

This pre‐defined sub‐study focused on patients with sepsis or septic shock who were included in the *International Point Prevalence Study of Intensive Care Unit Transfusion Practices* (InPUT) study.^24^

The InPUT‐study was an international, multicenter cohort study spanning 30 countries and 233 centers, focusing on ICU transfusion practices. A detailed protocol has been published previously.^25^

The study adhered to the Declaration of Helsinki and received institutional and ethical approvals. It was also conducted and reported in accordance with the Strengthening the Reporting of Observational Studies in Epidemiology (STROBE) guidelines, as outlined in Table S1.

### 2.2 Data collection

Data were prospectively collected over 16 pre‐scheduled weeks between March 2019 and October 2022, including all adult ICU patients admitted during this period (aged ≥18 years). This sub‐study focused on patients with sepsis or septic shock, including those who developed sepsis after ICU admission. These patients were followed daily until Day 28 or ICU discharge, whichever came first. Collected data included baseline demographics, daily clinical parameters (such as transfusion status and laboratory values), sequential organ failure assessment (SOFA) scores, and Day 28 outcomes.

Every administration of RBCs, platelets, or plasma was documented as a distinct transfusion event. In instances where multiple transfusions occurred within the same day, each was recorded separately. Laboratory findings and clinical indications were captured for each transfusion to provide insight into clinical decision‐making.

### 2.3 Definitions and outcomes

Sepsis was defined as a suspected or confirmed infection with an acute increase of ≥2 SOFA points. Septic shock was defined by the requirement for vasopressor therapy to maintain a mean arterial pressure (MAP) of ≥65 mmHg, along with a lactate level >2 mmol/L (18 mg/dL) despite adequate fluid resuscitation, as determined by the attending physician.^4^ Patients were classified as having developed sepsis if they were admitted for another reason and developed sepsis from Day 2 of ICU admission onward.

Anemia was classified according to World Health Organization guidelines, with Hb levels below 13 g/dL for males and below 12 g/dL for females.^26^ Thrombocytopenia was defined as platelet counts below 150 × 10^9^ cells/L.^27^ For descriptive purposes, an international normalized ratio (INR) >1.5 was considered elevated. Prophylactic transfusion was defined as administration of platelets or plasma intended to prevent spontaneous bleeding, excluding cases where transfusion was given in preparation for an invasive procedure, which were classified as pre‐procedure.

The study's primary outcomes were the incidence rates of blood component transfusions, including RBC, platelet, and plasma transfusions. Secondary outcomes included the clinical reasons for transfusions, comparisons of ICU length of stay, blood loss, and laboratory values between transfused and non‐transfused patients. Finally, mortality comparisons between transfusion groups were presented as unadjusted analyses. Given the complexity of potential confounders, performing adjusted analyses exceeded this study's scope. Supplemental analyses include statistical assessments focused on patients admitted in a state of septic shock.

### 2.4 Statistical analysis

Descriptive statistics were presented as mean (± standard deviation [SD]) or median (25th–75th percentile, interquartile range [IQR]) for continuous data, according to the data distribution. Categorical data were reported as counts and percentages (*N*, %). Transfusion events were documented as the overall occurrence rate and total units transfused for the entire sepsis cohort, as well as separate analyses focusing specifically on patients with septic shock (see supplementary analyses: Supplemental Table 2‐4). Analyses of Hb, platelet counts, or INR values were conducted only for transfusion events when these variables were reported. No data imputation was performed due to variable amounts of missing pre‐ or post‐transfusion data, which were assumed to be missing, not at random.

Statistical analyses included: (1) population comparisons based on transfusion status, and (2) in‐depth descriptive analyses for each specific transfusion type. To compare transfused and non‐transfused populations, either the Student's *t*‐test, Mann–Whitney *U* test, or chi‐squared test was used, where appropriate. A Bonferroni correction was applied to adjust for multiple pairwise comparisons within a single subset of data.

For all analyses, a two‐sided *p*‐value <.05 was considered significant. Differences are described using a Hodges–Lehmann estimated median, mean or percentage difference with 95% confidence interval. Data analyses were performed using R software within the RStudio interface (version 4.2.2; Boston, MA, 2022).

## 3 RESULTS

**FIGURE 1:**
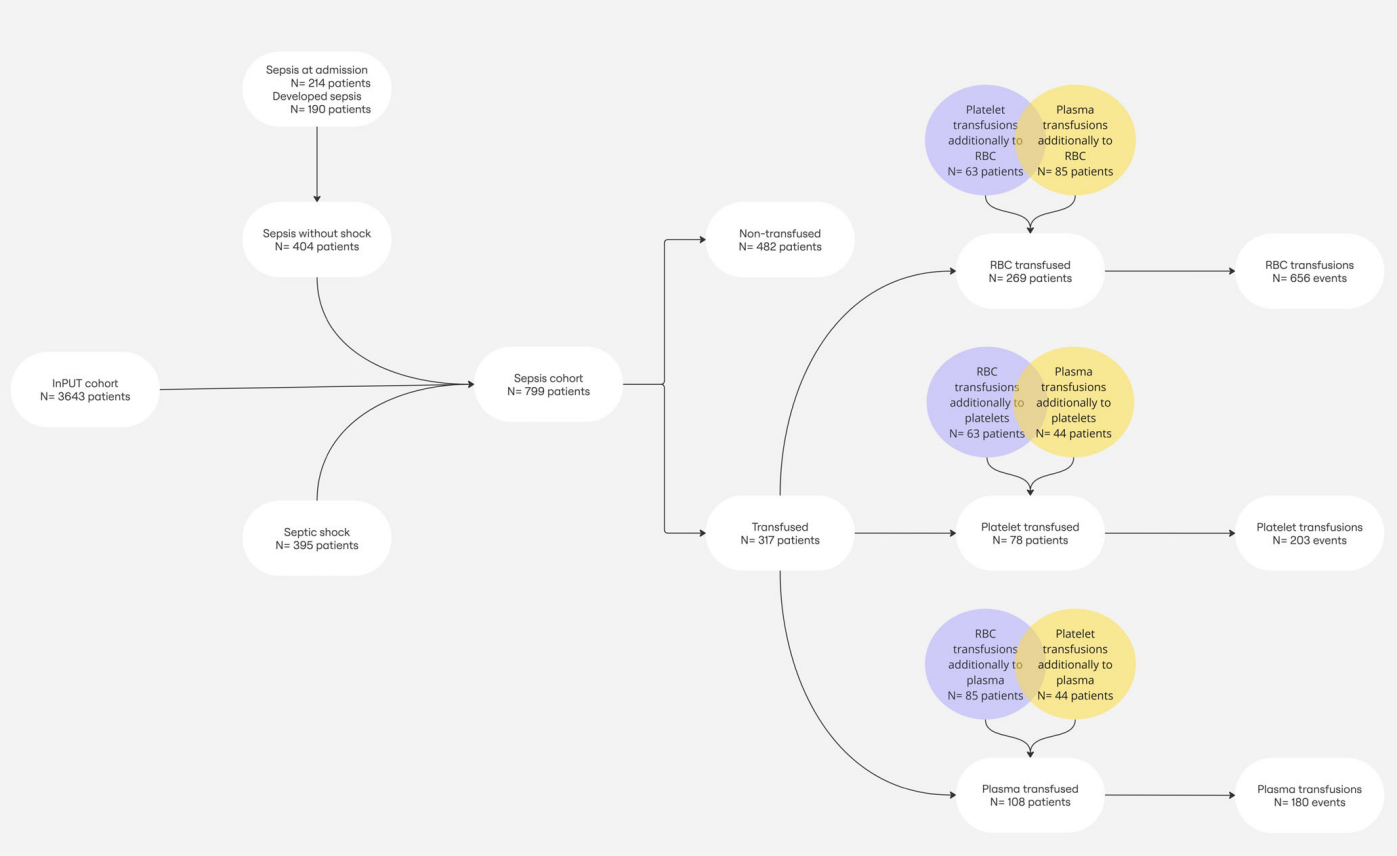
Study participants' pathway: from inclusion to transfusion events. RBC, red blood cell.

In this sub‐study, we included 799 patients who were admitted for sepsis, septic shock, or who developed sepsis subsequently during their ICU stay. This subset represented 22% of the total InPUT cohort (*N* = 3643). Within this sepsis cohort, 317 patients (40%, *N* = 317/799) received at least one type of blood product. Figure 1 shows a detailed overview of enrollment and transfusion status in this study.

Baseline characteristics of the study population are provided in Table 1. The majority of patients were male (61%, *N* = 491/799) with a median age of 65 years (IQR 55–75). Nine out of 10 admissions were in an emergency setting, with the primary referral source being the Emergency Room (36%, *N* = 293), followed by admissions from the hospital ward (31%, *N* = 249/799). Nearly half of the cohort (49%, *N* = 395) had septic shock at admission. Upon admission, 58% (*N* = 460/799) of the patients presented with anemia, 26% (*N* = 207/799) with thrombocytopenia, and 12% (*N* = 97/799) had an elevated INR above 1.5.

**TABLE 1:** Baseline characteristics of the study population, and stratified per transfusion status.

	All participants (*N* = 799)	Non‐transfused (*N* = 482)	Transfused (*N* = 317)	*p*‐Value	Difference^a^ 95% CI)
Patient characteristics					
Age	65 (55–75)	65 (56–75)	64 (55–75)	.60	1 (−2 to 3)
Female	308 (39)	187 (39)	121 (38)	.92	1 (−8 to 6)
Medical history^b^					
Acute coronary syndrome	71 (9)	43 (9)	28 (9)	1.0	0 (−4 to 4)
Chronic kidney disease	109 (14)	59 (12)	50 (16)	.2	3.5 (−1 to 9)
Chronic obstructive pulmonary disease	108 (14)	75 (16)	33 (10)	.58	5 (−1 to 10)
Heart failure	128 (16)	77 (16)	51 (16)	1.0	0 (−5 to 5)
Hematological disease—benign	11 (1)	7 (3)	4 (1)	1.0	0 (−1 to 2)
Hematological disease—malignant	39 (5)	14 (3)	25 (8)	.**03**	5 (2–8)
Liver failure	39 (5)	15 (3)	24 (8)	.00	5 (1–8)
Other	382 (48)	154 (32)	84 (27)	.1	6 (−1 to 12)
No comorbidities	222 (28)	135 (28)	87 (27)	.9	1 (−6 to 7)
Type of admission					
Emergency	711 (89)	438 (91)	273 (86)	.**05**	5 (0–9)
Presence of sepsis at admission, without shock	214 (27)	133 (28)	81 (25)	.56	2 (−4 to 8)
Presence of septic shock at admission	395 (49)	255 (53)	140 (44)	.**02**	9 (2–16)
Admitted for other reason (developed sepsis during admission)	190 (24)	94 (20)	96 (30)	**<.00**	11 (5–17)
Surgery <24 h prior to ICU admission	201 (25)	99 (21)	102 (32)	.**05**	12 (5–18)
Severity scores					
APACHE IV score at admission	64 (42–87)	60 (39–83)	72 (47–93)	**<.00**	10 (5–14)
SOFA score at first 24 h of sepsis diagnosis	7 (4–10)	7 (4–9)	8 (5–11)	**<.00**	2 (1–3)
Department prior to ICU admission					
Emergency room	293 (37)	196 (41)	97 (31)	**<.00**	10 (3–17)
Hospital ward	249 (31)	136 (28)	113 (36)	.**03**	7 (1–15)
Operating room	157 (20)	84 (17)	73 (23)	.06	6 (−0 to 11)
Other hospital	98 (12)	65 (14)	33 (10)	.24	3 (−2 to 8)
Other	2 (0)	1 (0)	1 (0)	1.0	0 (−1 to 1)
Additional supportive therapy at admission^b^					
Mechanical ventilation	380 (48)	219 (45)	161 (51)	.15	5 (−2 to 12)
Renal replacement therapy	75 (9)	37 (8)	38 (12)	.05	4 (0–9)
ECMO	5 (1)	0 (0)	5 (1)	.**01**	‐
Other support	23 (3)	11 (2)	12 (4)	.6	2 (−1 to 4)
Laboratory results (at admission)					
Hemoglobin (g/dL)	11.4 (±2.8)	12.3 (±2.5)	10.0 (±2.8)	**<.00**	2.3 (1.8–2.7)
Platelet count (×10^9^ cells/L)	202 (136–288)	208 (145–287)	189 (108–293)	.**02**	23 (3–42)
INR	1.2 (1.1–1.5)	1.2 (1.1–1.4)	1.4 (1.1–1.7)	**<.00**	0.1 (0–0.2)
Prothrombin time (s)	14 (13–17)	15 (13–17)	14 (13–17)	.9	0 (−2 to 3)
aPTT (s)	32 (28–38)	31 (28–36)	33 (28–42)	**<.00**	2 (1–4)
Hematological anomalies (at admission)					
Anemia	460 (58)	238 (49)	222 (70)	**<.00**	21 (14–27)
Thrombocytopenia	207 (26)	111 (23)	96 (30)	.**01**	7 (1–14)
Elevated INR (>1.5)	97 (12)	38 (8)	59 (19)	**<.00**	11 (6–16)
Elevated aPTT (>36 s)	135 (17)	62 (13)	73 (23)	**<.00**	10 (5–16)

*Note*: Data are expressed as mean ± standard deviation, median [25th–75th percentile], or number (%). Statistical comparisons were performed using the Student's *t*‐test, Mann–Whitney *U* test, or chi‐square test, with a significance threshold of *p* < .05 in bold. No Bonferroni correction was applied, results should be interpreted accordingly.

Abbreviations: APACHE IV, acute physiology and chronic health evaluation IV; aPTT, activated partial thromboplastin time; CI, confidence interval; ECMO, extracorporeal membrane oxygenation; h, hours; ICU, intensive care unit; INR, international normalized ratio; SOFA, sequential organ failure assessment.

^a^ Difference: Hodges Lehman median difference, mean difference or percentage difference with 95% confidence interval.

^b^ Multiple options possible.

### 3.1 RBC transfusion

**FIGURE 2:**
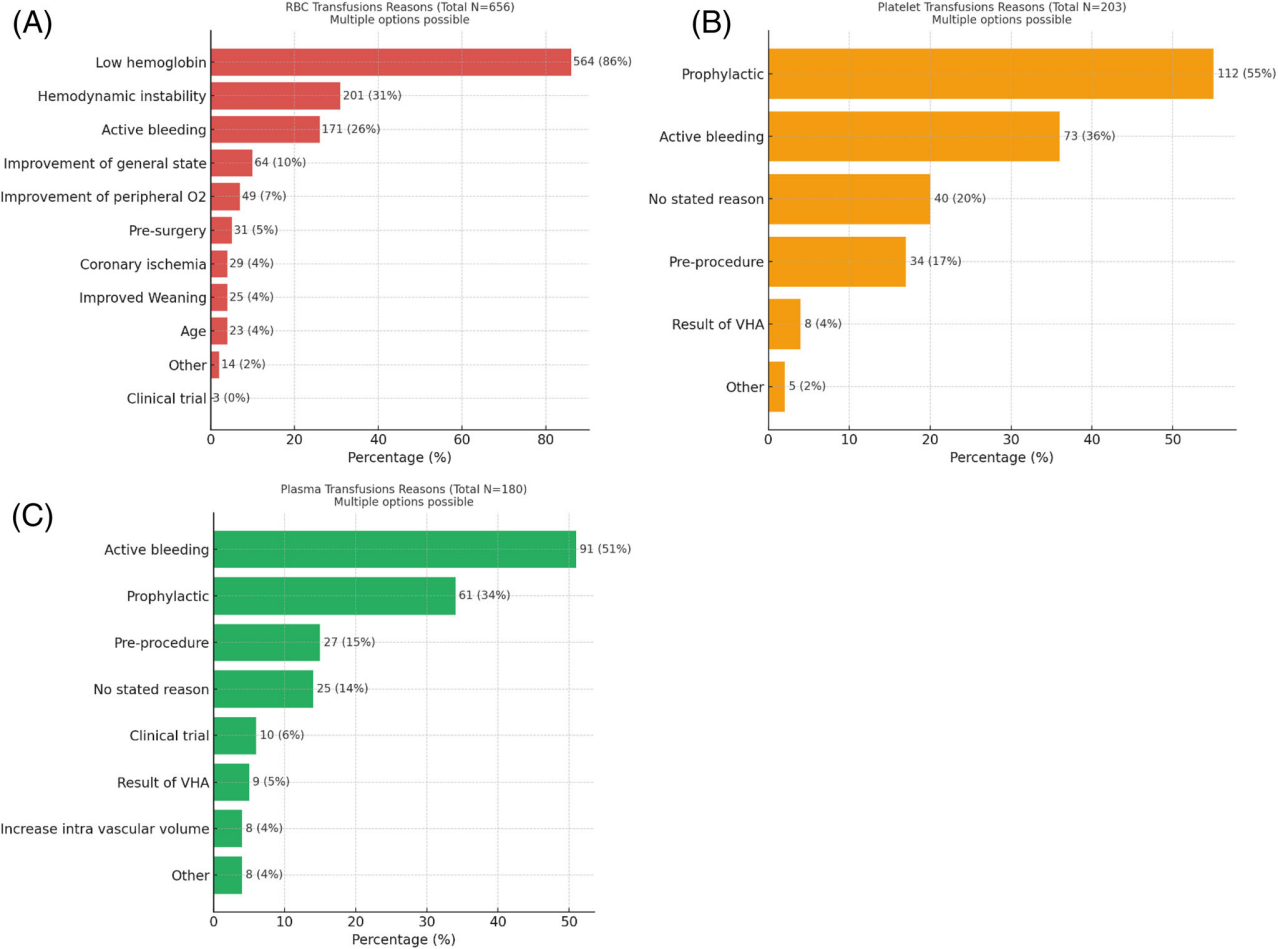
(A–C) Clinical reasons for red blood cell (RBC)/platelet/plasma transfusions multiple reasons for transfusion could be recorded per event. Other reasons included: Platelet transfusions;coagulopathy (*N* = 3), during surgery (*N* = 1), at the discretion of the hematology department (*N* = 1). Plasma transfusions;(low platelet count (*N* = 4), COVID‐19 (*N* = 3) (Coronavirus Disease 2019), low hemoglobin (*N* = 1). VHA, visoelastic hemostatic assay.

RBC transfusions were administered in 34% of patients (*N* = 269/799) during their ICU stay, with a median of two transfusion events per transfused patient (IQR 1–3), with a median of 2 units transfused per patient (IQR 1–5) (Table 2). Among the 656 RBC transfusion events (Figure 2A), low Hb was the predominant indication to transfuse (86%, *N* = 564/656), with additional common reasons being hemodynamic instability (31%, *N* = 201/656) and active bleeding (26%, *N* = 171/656). Overlap analyses showed that 258 events (39%) were due to low Hb alone, 165 (25%) to low Hb combined with hemodynamic instability, and 125 (19%) to low Hb combined with active bleeding. The mean Hb before transfusion was 7.5 ± 1.4 g/dL, which rose by 1.4 ± 1.3 g/dL post‐transfusion. One‐third of transfusions took place without a reported pre‐defined transfusion threshold, while only 23% of patients (*N* = 149/656) were assigned a restrictive threshold (<7 g/dL) prior to transfusion.

**TABLE 2:** Clinical characteristics of blood transfusion events.

RBC transfusions		
*N*. events	656	
*N*. of transfusion events per patient	2 (1–3)	
Total units transfused per patient	2 (1–5)	
*N*. RBC units per event	1 (1–2)	
Product ordered by		
Intensivist	464 (71)	
Specialist, non‐intensivist	22 (3)	
Resident	94 (14)	
Other	76 (12)	
Primary medical specialty of transfusion requestor		
Anesthesiology	316 (48)	
Intensivist	149 (24)	
Cardiology	9 (1)	
Internal medicine	116 (18)	
Pulmonology	32 (5)	
Surgery	29 (4)	
Other	4 (1)	
Hemoglobin values		
Hb measured prior to transfusion event	629 (96)	
Hb before transfusion (g/dL)	7.5 (±1.4)	
Hb post‐transfusion (g/dL)	8.9 (±1.3)	
Hb increase after transfusion (g/dL)	1.4 (±1.3)	
Pre‐defined threshold (g/dL)	8 (7–9)	
Transfusion policy	Stated target	Used target
Restrictive (<7 g/dL)	149 (23)	232 (35)
Intermediate (7–9 g/dL)	157 (24)	323 (49)
Liberal (>9 g/dL)	137 (21)	56 (9)
No threshold	213 (33)	45 (7)
*N*. MTP	13 (2)	
*N*. whole blood	6 (1)	

Platelet transfusion		
*N*. events	203	
*N*. of transfusion events per patient	1 (1–2)	
Total units transfused per patient	2 (1–6)	
*N*. platelet units per event	1 (1–2)	
Product ordered by		
Intensivist	149 (73)	
Specialist, non‐intensivist	34 (17)	
Resident	17 (8)	
Other	3 (2)	
Primary medical specialty of transfusion requestor		
Anesthesiology	64 (32)	
Intensivist	44 (22)	
Cardiology	2 (1)	
Internal medicine	78 (39)	
Pulmonology	2 (1)	
Surgery	8 (4)	
Other	3 (2)	
Platelet values		
Platelets measured prior to transfusion event	184 (91)	
Platelet count before transfusion (×10^9^ cells/L)	26 (16–51)	
Platelet count post‐transfusion (×10^9^ cells/L)	54 (27–78)	
Platelet count increase (×10^9^ cells/L)	15 (4–32)	
Transfusion policy	Stated target	Used target
Minimal (≤20 × 10^9^ cells/L)	21 (10)	65 (32)
Low (21–50 × 10^9^ cells/L)	62 (31)	71 (35)
Intermediate (51–100 × 10^9^ cells/L)	12 (6)	37 (18)
High (>100 × 10^9^ cells/L)	12 (6)	10 (5)
No threshold	96 (47)	19 (9)
Antiplatelet use in prior week	12 (6)	

Plasma transfusion		
*N*. events	180	
*N*. of transfusion events per patient	1 (1–2)	
Total units transfused per patient	3 (2–5)	
*N*. plasma units per event	2 (1–3)	
Product ordered by		
Intensivist	136 (76)	
Specialist, non‐intensivist	30 (17)	
Resident	14 (8)	
Other	0 (0)	
Primary medical specialty of transfusion requestor		
Anesthesiology	104 (58)	
Intensivist	25 (14)	
Cardiology	5 (3)	
Internal medicine	26 (14)	
Pulmonology	9 (5)	
Surgery	10 (6)	
Other	1 (1)	
Lab values		
INR measured before transfusion	152 (84)	
INR target	1.3 (1.2–1.5)	
*N*. INR target stated	107 (59)	
INR before transfusion	1.6 (1.3–2.4)	
INR after transfusion	1.5 (1.2–2.0)	
INR decrease after transfusion	0.2 (0.0–0.6)	
Transfusion policy	Stated target	Used target
INR >3.0	0 (0)	26 (14)
INR 1.5–3.0	21 (12)	56 (31)
INR <1.5	86 (48)	70 (39)
No threshold	73 (41)	28 (16)
Anticoagulant use in week before transfusion	75 (42)	

*Note*: Data are presented as mean ± standard deviation (SD), median [25th–75th percentile], or count (%). “Stated target” indicates the intended transfusion threshold as reported by the treating clinician, whereas “Used target” denotes the actual laboratory value (Hb, platelet count, or INR) at the time of transfusion.

Abbreviations: Hb, hemoglobin; ICU, intensive care unit; INR, international normalized ratio; MTP, massive transfusion protocol; RBC, red blood cells.

**FIGURE 3:**
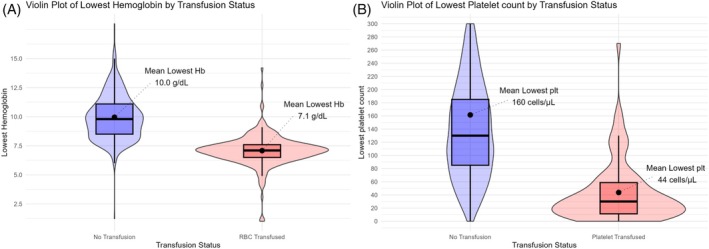
(A) A violin plot with boxplot overlay illustrating the distribution of minimum hemoglobin (Hb) levels by transfusion status, with the red blood cell transfused group shown in red and the non‐transfused group in blue. (B) A violin plot with boxplot overlay illustrating the distribution of minimum platelet count levels by transfusion status, with the platelet‐transfused group shown in red and the nontransfused group in blue. Abbreviations: RBC, red blood cell; plt, platelet count.

At admission, 58% of patients with sepsis or septic shock (*N* = 460/799) were anemic; by the end of the study period, this proportion rose to 93% (*N* = 744/799) (Table 3). Nearly all who received RBC transfusions (99%, *N* = 266/269) experienced anemia during their ICU stay. Compared to non‐transfused patients, those receiving RBCs showed significantly lower mean weighted Hb levels and nadir values; as illustrated in Figure 3A, mean lowest Hb was 7.1 ± 1.4 g/dL among transfused patients versus 10.0 ± 1.9 g/dL in non‐transfused patients. RBC‐transfused patients also showed a higher mean SOFA score throughout their ICU stay and a longer median ICU stay compared to non‐transfused patients (Table 3). By Day 28, overall mortality was 39% (*N* = 313/799), with unadjusted mortality rates of 42% (*N* = 222/530) in transfused and 34% (*N* = 91/269) in non‐transfused patients.

**TABLE 3:** Detailed clinical characteristics during intensive care unit stay, stratified by transfusion status.

	All participants	Non‐transfused	Transfused	*p*‐Value	Difference (95% CI)^a^
Red blood cell transfusion					
*N*. patients	799 (100)	530 (66)	269 (34)		
Median ICU stay (days)	5 (3–11)	5 (3–9)	8 (4–16)	**<.00**	3 (2–3)
Blood loss (mL)^b^	40 (±120)	15 (±58)	88 (±182)	**<.00**	72 (49–94)
SOFA^b^	6.6 (±4)	6.2 (±4)	7.6 (±4)	**<.00**	1.4 (0.8–2.1)
Laboratory values during ICU stay					
Hb^b^	10 (±2.0)	10.8 (±1.9)	8.6 (±1.3)	**<.00**	2.3 (2.0–2.5)
Nadir Hb	8.9 (±2.2)	10.0 (±1.9)	7.1 (±1.4)	**<.00**	2.9 (2.7–3.1)
Anemia at admission	460 (58)	266 (50)	194 (72)	**<.00**	22 (15–29)
Anemia^c^	744 (93)	478 (90)	266 (99)	**<.00**	9 (6–12)
Platelet transfusion					
*N*. patients	799 (100)	721 (90)	78 (10)		
Median ICU stay (days)	5 (3–11)	5 (3–10)	8 (4–18)	**<.00**	2 (1–3)
Blood loss (mL)^b^	40 (±120)	32 (±106)	110 (±197)	**<.00**	78 (33–123)
SOFA^b^	6.6 (±3.9)	6.3 (±3.7)	10.1 (±4.5)	**<.00**	3.8 (2.7–5)
Laboratory results during ICU stay					
Platelet count^b^	197 (±120)	210 (±117)	77 (±77)	**<.00**	133 (109–157)
Nadir platelet count	126 (68–195)	138 (86–203)	23 (10–42)	**<.00**	120 (105–136)
Admission thrombocytopenia	207 (26)	160 (22)	47 (60)	**<.00**	38 (27–49)
Thrombocytopenia^d^	453 (57)	378 (52)	75 (96)	**<.00**	44 (38–49)
51–150 × 10^9^ cells/L	326 (41)	306 (42)	20 (26)	**<.00**	17 (6–27)
21–50 × 10^9^ cells/L	85 (11)	57 (8)	28 (36)	**<.00**	28 (17–39)
≤20 × 10^9^ cells/L	42 (5)	15 (2)	27 (35)	**<.00**	33 (22–43)
Plasma transfusion					
*N*. patients	799 (100)	691 (87)	108 (13)		
Median ICU stay (days)	5 (3–11)	5 (3–10)	8 (4–16)	**<.00**	2 (1–3)
Blood loss (mL)^b^	40 (120)	24 (78)	139 (240)	**<.00**	115 (69–161)
SOFA^b^	6.6 (±3.9)	6.2 (±3.6)	9.3 (±4.7)	**<.00**	3 (2–4)
Admission INR elevated >1.5	97 (12)	72 (10)	25 (23)	**.02**	13 (5–21)

*Note*: Data are presented as mean ± standard deviation (SD), median [25th–75th percentile], or count (%). Group differences were evaluated using Student's *t*‐test, Mann–Whitney *U* test, chi‐square test, Analysis of Variance (ANOVA), or Kruskal–Wallis test with Bonferroni correction, with significance defined as *p* < .05 indicated in bold.

Abbreviations: CI, confidence interval; Hb, hemoglobin; ICU, intensive care unit; INR, international normalized ratio; SOFA, sequential organ failure assessment.

^a^ Difference: Hodges Lehman median difference, mean difference and percentage difference with 95% confidence interval.

^b^ Weighted by ICU length of stay (days).

^c^ Anemia defined as hemoglobin <12 g/dL for women and <13 g/dL for men (according to definition World Health Organization).

^d^ Thrombocytopenia defined as platelet count <150 cells × 10^9^/L, during ICU stay.

### 3.2 Platelet transfusion events

Platelet transfusions occurred in 10% of patients (*N* = 78/799) during their ICU stay, with a median of 1 transfusion event per transfused patient (IQR 1–2). As shown in Figure 2B, prophylactic transfusions were the most frequent indication (55%, *N* = 112/203), followed by transfusions for active bleeding (36%, *N* = 73/203). Pre‐transfusion platelet counts were available in 91% of transfusion events (*N* = 184/203), with a median of 26 × 10^9^ cells/L (IQR 16–51 × 10^9^ cells/L). Post‐transfusion platelet counts were available in 62% of events (*N* = 125/203), with a median increase of 15 × 10^9^ cells/L (IQR 4–32 × 10^9^ cells/L) after transfusion.

Nearly half of the platelet‐transfused patients had no pre‐defined transfusion threshold. Among those who received platelet transfusions, 96% (*N* = 75/78) developed thrombocytopenia at some point during ICU admission; in contrast, 22% (*N* = 160/799) of non‐transfused patients had thrombocytopenia at admission, rising to 52% (*N* = 378/799) by the end of the study period (Table 3). Transfused patients had significantly lower nadir platelet counts than non‐transfused patients (23 × 10^9^ cells/L [IQR 10–42 × 10^9^ cells/L] vs. 138 × 10^9^ cells/L [IQR 86–203 × 10^9^ cells/L]; Figure 3B).

These patients also frequently required other blood products: 81% received RBCs and 56% received plasma (Figure 1). They had higher weighted mean SOFA scores (10.1 ± 4.5 vs. 6.3 ± 3.7), longer ICU stays, and greater blood loss (110 ± 197 mL vs. 32 ± 106 mL). Reflecting their greater illness severity, unadjusted 28‐day mortality was higher in transfused patients (60%, *N* = 47/78) than in non‐transfused patients (34%, *N* = 245/721).

### 3.3 Plasma transfusion events

Plasma transfusions were administered to 108 (14%) patients, totaling 180 transfusions, with a median of one transfusion event per patient (IQR 1–2) and 3 units per event (IQR 2–5) (Table 2). Pre‐transfusion INR was recorded in 84% of these events, with a median of 1.6 (IQR 1.3–2.4), decreasing by 0.2 (IQR 0.0–0.6) following transfusion. In 41% of transfusions (*N* = 74/180), no specific INR target was documented prior to transfusion. Figure 2C shows active bleeding as the most frequent clinical indication (51%, *N* = 91/180), followed by prophylactic treatment (34%, *N* = 61/180).

Evaluated independently, 23% (*N* = 25/108) of the plasma recipients had an INR over 1.5 upon admission, in contrast to 10% (*N* = 72/691) of those who did not receive plasma. While pre‐transfusion INR was measured in most transfusion events, under 5% of patients underwent routine prothrombin time (PT) or INR checks during their ICU stay. A substantial number of plasma‐transfused patients additionally received other blood products, with 79% (*N* = 85/108) receiving RBCs and 41% (*N* = 44/108) receiving platelets (Figure 1).

Plasma recipients were more critically ill, reflected in higher SOFA scores (9.3 ± 4.7 vs. 6.2 ± 3.6), greater blood loss (139 ± 240 mL vs. 24 ± 78 mL), and longer ICU stays (median 8 days [IQR 4–16] vs. 5 days [IQR 3–10]). In line with these findings, unadjusted 28‐day mortality was higher among plasma‐transfused patients (51%, *N* = 55/108) than in the non‐transfused group (35%, *N* = 241/691).

## 4 DISCUSSION

This study represents the largest prospective, international, multicenter observational cohort investigating contemporary transfusion practices in ICU patients with sepsis. In this population, 40% of patients underwent transfusion with at least one type of blood product. One‐third (34%) of patients received RBC transfusions, primarily to manage low Hb levels and establish hemodynamic instability, with Hb thresholds broadly aligning with current transfusion guidelines. Platelet transfusions were administered to approximately 10% of patients, while plasma transfusions were given to 14%, mainly for prophylactic purposes or to treat active bleeding.

RBC transfusions continue to play a pivotal role in managing sepsis, as shown by their use in one‐third of patients in this cohort. Current guidelines and literature recommend reserving RBC transfusions for sepsis patients with a Hb below 7 g/dL, regardless of hemodynamic state.^1^, ^9^, ^10^

Our mean pre‐transfusion Hb (7.5 ± 1.4 g/dL) aligns with a 2019 ICU physician survey,^22^ indicating improved compliance with restrictive transfusion guidelines compared to a transfusion‐related sub‐analysis of the Intensive Care Over Nations (ICON) cohort (8.1 ± 1.5 g/dL). The ICON study was a worldwide observational cohort study of general ICU patients conducted in 2012.^2^ However, given that transfusion was not the primary endpoint in the ICON study, a degree of reporting bias cannot be ruled out. Patient‐specific variables and clinical judgment remain paramount, indicating that transfusion decisions should not be reduced to a single rigid cut‐off.

Platelet and plasma transfusions were less common than RBC transfusions. Platelet transfusions were administered to 10% (*N* = 78/799) of patients, either to mitigate bleeding risk or to control acute bleeding. Thrombocytopenia, a common condition among ICU patients, is particularly prevalent and severe in those with sepsis, who face elevated bleeding risks and often exhibit significantly lower platelet counts compared to the general ICU population.^28^, ^29^, ^30^ This heightened risk is driven by sepsis‐induced coagulation system activation, coupled with suppression of anticoagulation and fibrinolytic pathways, which promote microthrombi formation and further reduce platelet counts.^31^, ^32^

Thrombocytopenia is increasingly recognized as a marker of poor prognosis in sepsis, often reflecting systemic inflammation, coagulopathy, and organ dysfunction.^33^ While platelet transfusions have traditionally been administered to reduce bleeding risk prior to invasive procedures, this practice is being increasingly challenged by a lack of supporting evidence.^1^, ^17^, ^34^, ^35^ Indeed, a recent meta‐analysis performed for the international guideline from the Advancement of Blood and Biotherapies (AABB) demonstrated that restrictive platelet transfusion strategies were non‐inferior to liberal ones, leading to a lowering of thresholds in different procedural and prophylactic contexts.^36^ Within the platelet‐transfused cohort, most transfusions were administered either for prophylactic purposes or prior to invasive procedures. This practice underscores the need to adhere to evidence‐based recommendations and to pursue additional research, as emerging data increasingly question traditional liberal thresholds and the overall efficacy and safety of platelet transfusions in this setting.

In our cohort, 14% (*N* = 108/799) of patients received plasma transfusions, split equally between bleeding and non‐bleeding indications. This contrasts with prior reports indicating that plasma is frequently administered or considered in over half of non‐bleeding sepsis patients,^22^, ^37^ which may reflect a shift in clinical practice given the limited supporting evidence.^1^

Despite these trends, sepsis‐induced coagulopathy (SIC) remains a significant challenge, affecting 50% to 70% of sepsis patients and progressing to DIC in about 30%.^38^, ^39^ There is no evidence supporting plasma transfusions for managing SIC or DIC; current evidence suggests that plasma transfusions do not improve coagulopathy and are associated with increased mortality, particularly in patients with sepsis.^15^, ^40^ Therefore, the administration of plasma for non‐bleeding patients in our study contrasts with current guidelines, which discourage plasma transfusions in these patients.^1^ Moreover, studies consistently report no benefit from prophylactic plasma transfusions, either in the general ICU population,^41^, ^42^, ^43^, ^44^ or specifically among sepsis patients.^15^, ^20^, ^45^ These transfusions frequently fail to achieve meaningful hemostatic improvements and may pose additional risks, including volume overload, immune modulation, and an increased susceptibility to infections.^46^ These results call for further research to identify alternative strategies for managing coagulation abnormalities in septic patients, focusing on interventions that balance efficacy and safety.

### 4.1 Strengths and limitations

This study provides several strengths that contribute to a deeper understanding of transfusion practices in ICU patients with sepsis. As one of the most extensive prospective cohort studies focusing on sepsis, it evaluates transfusion practices across diverse healthcare settings, offering a robust and globally representative depiction of current practices. Additionally, the prospective design ensured systematic data collection, reducing the risk of recall bias and increasing the reliability of our results. Including specific reasons for transfusion decisions further adds depth to the analyses, providing nuanced insights into the use of various blood products in patients with sepsis.

While this study provides valuable insights into transfusion practices in ICU patients with sepsis, it is not without limitations. Despite its international scope, most centers were in middle‐ to high‐income countries, limiting generalizability to lower‐income settings. A notable limitation was the inability to monitor the transition from sepsis to septic shock during the ICU stay, as daily lactate measurements were not included in the study protocol. Consequently, we were only able to identify patients admitted in a state of septic shock, potentially underestimating the actual burden of septic shock in this cohort. Further, the absence of data on the source of sepsis limits our understanding of how different infection etiologies may impact transfusion practices. Similarly, the lack of fluid balance data, which influences both hemodynamic stability and Hb concentrations, limits our ability to fully contextualize transfusion thresholds within the broader framework of sepsis management.

Finally, mortality comparisons between transfusion groups were presented as unadjusted analyses. Given the complexity of potential confounders, performing adjusted analyses exceeded this study's scope. Consequently, these comparisons should be interpreted cautiously and cannot be regarded as establishing causal relationships between transfusions and mortality.

In conclusion, this study provides a comprehensive overview of transfusion practices in patients with sepsis and septic shock, demonstrating frequent use of RBC, platelet, and plasma transfusions. While RBC transfusions largely follow restrictive thresholds in line with evidence‐based guidelines, platelet and plasma transfusions are often administered for indications with limited supporting evidence. These findings highlight areas for future research and potential opportunities to improve clinical practice.

## CONFLICT OF INTEREST STATEMENT

Dr. Cecconi reported receiving personal fees from Edwards Lifesciences, GE Healthcare, and Directed Systems outside the submitted work. Dr. Shah reported receiving consultancy fees from Pharmacosmos UK outside of the submitted work. Dr. Feldheiser reported receiving personal fees from Baxter and Medtronic outside the submitted work. Dr. Scheeren reported serving as senior medical director for Edwards Lifesciences (Garching, Germany). Dr. McQuilten reported receiving grants from Australian National Blood Authority and National Health and Medical Research Council during the conduct of the study. Dr. Flint reported receiving grants from the Australian National Blood Authority and Blood Synergy (Monash University) during the conduct of the study. Dr. Piagnerelli reported receiving grants from Centre Federal d'Expertise Belge–KCE grant for COVID‐19 study outside the submitted work. Dr. Gurjar reported receiving royalties for edited books (Manual of ICU Procedures and Textbook of Ventilation, Fluids, Electrolytes and Blood Gases) from the publisher Jaypee Brothers Medical Publishers (Pvt) Ltd., New Delhi. Dr. Pfortmueller reported receiving grants from Orion Pharma, Abbott Nutrition International, B Braun Medical AG, CSEM AG, Edwards Lifesciences Services GmbH, Kenta Biotech Ltd., Maquet Critical Care AB, Omnicare Clinical Research AG, Nestle, Pierre Fabre Pharma AG, Pfizer, Bard Medica SA, Abbott AG, Anandic Medical Systems, Pan Gas AG Healthcare, Bracco, Hamilton Medical AG, Fresenius Kabi, Getinge Group Maquet AG, Dräger AG, Teleflex Medical GmbH, GlaxoSmithKline, Merck Sharp and Dohme AG, Eli Lilly and Co, Baxter, Boehringer Ingelheim, Aseptuva, Astellas, AstraZeneca, CSL Behring, Novartis, Covidien, and Nycomed outside the submitted work; the funds were paid into departmental funds and no personal financial gain applied. Dr. Nielsen reported receiving personal fees from Adrenomed outside the submitted work. Dr. Vlaar reported receiving personal fees from a Vidi grant (ZonMW: 09150172010047). No other disclosures were reported.

## Supporting information


**Data S1.** Supporting Information.

## Data Availability

The data that support the findings of this study are available on request from the corresponding author. The data are not publicly available due to privacy or ethical restrictions.
