# Supplementary material for: Global transfusion practices in septic patients in the intensive care unit: insights from the InPUT‐study sub‐analysis
Source: Transfusion. 2025 Oct 22;65(12):2272–85. doi: 10.1111/trf.18445 (PMC12704699; doi:10.1111/trf.18445)
Supplement: Supplementary file 1 — Data S1. Supporting Information. [file TRF-65-2272-s001.pdf]

Supplemental Table 1. STROBE guidelines checklist

STROBE Statement—checklist of items that should be included in reports of observational studies

	Item No	Recommendation	Page N.
**Title and abstract**	1	(*a*) Indicate the study's design with a commonly used term in the title or the abstract	*p. 1*
		(*b*) Provide in the abstract an informative and balanced summary of what was done and what was found	*p. 4*
**Introduction**			
Background/rationale	2	Explain the scientific background and rationale for the investigation being reported	*p. 5/6*
Objectives	3	State specific objectives, including any prespecified hypotheses	*p. 5/6*
**Methods**			
Study design	4	Present key elements of study design early in the paper	*p. 6*
Setting	5	Describe the setting, locations, and relevant dates, including periods of recruitment, exposure, follow-up, and data collection	*p. 6*
Participants	6	(*a*) *Cohort study*—Give the eligibility criteria, and the sources and methods of selection of participants. Describe methods of follow-up<br>*Case-control study*—Give the eligibility criteria, and the sources and methods of case ascertainment and control selection. Give the rationale for the choice of cases and controls<br>*Cross-sectional study*—Give the eligibility criteria, and the sources and methods of selection of participants	*p. 6*
		(*b*) *Cohort study*—For matched studies, give matching criteria and number of exposed and unexposed<br>*Case-control study*—For matched studies, give matching criteria and the number of controls per case	*n/a*
Variables	7	Clearly define all outcomes, exposures, predictors, potential confounders, and effect modifiers. Give diagnostic criteria, if applicable	*p. 6/7*
Data sources/ measurement	8*	For each variable of interest, give sources of data and details of methods of assessment (measurement). Describe comparability of assessment methods if there is more than one group	*p. 6/7*

Bias	9	Describe any efforts to address potential sources of bias	*p. 7*
Study size	10	Explain how the study size was arrived at	*p. 6*
Quantitative variables	11	Explain how quantitative variables were handled in the analyses. If applicable, describe which groupings were chosen and why	*p. 7*
Statistical methods	12	(*a*) Describe all statistical methods, including those used to control for confounding	*p. 7*
		(*b*) Describe any methods used to examine subgroups and interactions	*p. 7*
		(*c*) Explain how missing data were addressed	*p. 7*
		(*d*) *Cohort study*—If applicable, explain how loss to follow-up was addressed<br>*Case-control study*—If applicable, explain how matching of cases and controls was addressed<br>*Cross-sectional study*—If applicable, describe analytical methods taking account of sampling strategy	*p. 7*
		(*e*) Describe any sensitivity analyses	*p. 7*

Results

Participants	13*	(a) Report numbers of individuals at each stage of study—eg numbers potentially eligible, examined for eligibility, confirmed eligible, included in the study, completing follow-up, and analysed	p. 8 & Figure 1
		(b) Give reasons for non-participation at each stage	p. 8 & Figure 1
		(c) Consider use of a flow diagram	*Figure 1*
Descriptive data	14*	(a) Give characteristics of study participants (eg demographic, clinical, social) and information on exposures and potential confounders	*Table 1 p. 13*
		(b) Indicate number of participants with missing data for each variable of interest	Supplemental Table & figures p.11 – p.17
		(c) *Cohort study*—Summarise follow-up time (eg, average and total amount)	*p. 8/9*
Outcome data	15*	*Cohort study*—Report numbers of outcome events or summary measures over time	Table 2 & 3, p. 15-17

		Case-control study—Report numbers in each exposure category, or summary measures of exposure	-
		Cross-sectional study—Report numbers of outcome events or summary measures	-
Main results	16	(*a*) Give unadjusted estimates and, if applicable, confounder-adjusted estimates and their precision (eg, 95% confidence interval). Make clear which confounders were adjusted for and why they were included	*Table 1-3, p. 15-18*
		(*b*) Report category boundaries when continuous variables were categorized	*Table 1-3, p. 15-18*
		(*c*) If relevant, consider translating estimates of relative risk into absolute risk for a meaningful time period	-
Other analyses	17	Report other analyses done—eg analyses of subgroups and interactions, and sensitivity analyses	-
**Discussion**			
Key results	18	Summarise key results with reference to study objectives	*p. 10*
Limitations	19	Discuss limitations of the study, taking into account sources of potential bias or imprecision. Discuss both direction and magnitude of any potential bias	*p. 11/12*
Interpretation	20	Give a cautious overall interpretation of results considering objectives, limitations, multiplicity of analyses, results from similar studies, and other relevant evidence	*p. 11/12*
Generalisability	21	Discuss the generalisability (external validity) of the study results	*p. 10/11*
**Other information**			
Funding	22	Give the source of funding and the role of the funders for the present study and, if applicable, for the original study on which the present article is based	-

*Give information separately for cases and controls in case-control studies and, if applicable, for exposed and unexposed groups in cohort and cross-sectional studies.

Supplemental Table 2. Baseline characteristics of the septic shock cohort, and stratified per transfusion status

	**All Septic shock participants** (n = 395)	**Non-transfused** (N= 255)	**Transfused** (N= 140)	***p*-value**
**Patient characteristics**				
Age median	66 (56 – 75)	66 (57 – 75)	66 (55 – 75)	0.95
Female	174 (44)	112 (44)	62 (44)	1.0
Medical history				
Acute coronary syndrome	32 (8)	21 (8)	11 (8)	1.0
Benign hematological disease	6 (2)	4 (2)	2 (1)	1.0
Chronic kidney disease	52 (13)	32 (13)	20 (14)	1.0
Chronic obstructive pulmonary disease	56 (14)	39 (15)	17 (12)	1.0
Heart failure	62 (16)	40 (16)	22 (16)	1.0
Malign hematological disease	19 (5)	10 (4)	9 (6)	0.4
Liver failure	24 (6)	7 (3)	17 (12)	**0.00**

No comorbidities	117 (30)	80 (31)	37 (26)	0.1
**Type of admission**				
Emergency	370 (93.7)	241 (94.1)	129 (92.1)	0.47
Surgery <24 h prior to ICU admission	110 (28)	59 (23)	51 (36)	**0.00**
**Severity scores**				
APACHE IV score at admission	70 (48 – 93)	65 (45 – 88)	82 (59 – 107)	**0.00**
SOFA score at first 24h of sepsis diagnosis	7 (5 – 11)	7 (5 – 10)	8 (6 – 12)	**0.00**
**Department prior to ICU admission**				
Emergency room	175 (44.3)	121 (47.5)	54 (38.6)	**0.00**
Hospital ward	104 (26.3)	61 (23.9)	43 (30.7)	0.3
Operating room	82 (20.8)	48 (18.8)	34 (24.3)	0.5
Other hospital	33 (8.4)	25 (9.8)	8 (5.7)	**0.02**
**Additional supportive therapy at admission**				
Mechanical ventilation	179 (45.3)	113 (44.3)	66 (47.1)	0.06
Renal replacement therapy	44 (11.1)	44 (11.1)	44 (11.1)	0.1
ECMO	2 (0.5)	2 (0.5)	2 (0.5)	0.24
No support	330 (83.5)	330 (83.5)	330 (83.5)	0.07
**Laboratory results (at admission)**				
Hemoglobin (g/dL)	11.3 (9.4 – 13.4)	12.2 (10.4 – 14.1)	9.5 (8.0 – 12.0)	**0.00**
Platelet count (x10^3/µL)	200 (132 – 287)	204 (144 – 282)	186 (98 – 296)	0.08
INR	1.3 (1.2 – 1.5)	1.2 (1.1 – 1.4)	1.4 (1.2 – 1.7)	**0.01**
Prothrombin time (sec)	14.8 (12.9 – 17.8)	14.1 (12.7 – 16.7)	22.6 (18 – 25.8)	**0.1**
aPTT (sec)	32.4 (28.5 – 38.7)	32 (28.2 – 36.3)	34.8 (29 – 42)	0.05

**Hematological anomalies at admission**				
Anemia	235 (59.5)	129 (50.5)	106 (75.7)	**0.00**
Thrombocytopenia	113 (28.6)	62 (24.3)	51 (36.4)	**0.03**
N. elevated INR (>1.5)	51 (12.9)	21 (8.2)	30 (21.3)	**0.00**
N. elevated APTT (>36 sec)	75 (19.0)	39 (15.3)	36 (25.7)	**0.01**

Data are expressed as mean ± standard deviation, median [25th–75th percentile], or number (%). Statistical comparisons were performed using the Student's t-test, Mann–Whitney U test, or Chi-square test, with a significance threshold of ***p<0.05****. No Bonferroni correction was applied, results should be interpreted accordingly.* **: multiple options possible.

Abbreviations: *APACHE IV – Acute Physiology and Chronic Health Evaluation IV; aPTT – Activated Partial Thromboplastin Time; ECMO – Extracorporeal Membrane Oxygenation; ICU – Intensive Care Unit; INR – International Normalized Ratio; PT – Prothrombin Time; RBC – Red Blood Cells; SOFA – Sequential Organ Failure Assessment.*

Supplement Table 2. Clinical Characteristics of Blood Transfusion Events in Septic shock cohort

**RBC transfusions**		
N. events	242	
N. of transfusion events per patient	2 (1 – 3)	
Total units transfused per patient	4 (2 – 8)	
N. RBC units per event	2 (1 – 4)	
Product ordered by (%)		
Intensivist	167 (69)	
Specialist, non-intensivist	7	
Resident	41	
Other	27	
Primary medical specialty of transfusion requestor		
Anesthesiology	95	
Intensivist	68	
Cardiology	5	
Internal medicine	52	
Pulmonology	8	
Surgery	12	
Other	1	
Hemoglobin values		
Hb measured prior to transfusion event	228 (94)	
Hb before transfusion (g/dL)	7.3 (± 1.3)	
Hb post transfusion (g/dL)	8.7 (± 1.2)	
Hb increase after transfusion	1.4 (± 1.3)	
Pre-defined threshold (g/dL)	8 (7 – 9)	
Transfusion policy	*Stated target*	*Used target*
Restrictive (<7 g/dL)	57 (23.6)	94 (38.8)

Intermediate (7-9 g/dL)	57 (23.6)	117 (48.3)
Liberal (>9 g/dL)	47 (19.4)	15 (6.2)
No threshold	81 (33.5)	16 (6.6)
N. MTP	5 (2.1)	
N. Wholeblood	1 (0.4)	
**Platelet transfusion**		

N. events	98
N. of transfusion events per patient	1 (1 – 3)
Total units transfused per patient	2 (1 – 5)
N. platelet units per event	1 (1 – 2)
Product ordered by (%)	
Intensivist	67 (68.4)
Specialist, non-intensivist	3 (3.1)
Resident	11 (11.2)
Other	17
Primary medical specialty of transfusion requestor	
Anesthesiology	39 (39.8)
Intensivist	18 (18.4)
Cardiology	1 (1)
Internal medicine	31 (31.6)
Pulmonology	2 (2.0)
Surgery	6 (6.1)
Other	1 (1.0)
Platelet values	
Platelet count before transfusion ($10^3/\mu L$)	24 (13 – 38)
Platelet count post transfusion ($10^3/\mu L$)	50 (25 – 70)

Platelet count increase after transfusion (10^3/µL)	15 (3 – 32)	
Transfusion policy (%)	*Stated target*	*Used target*
Minimal (≤20 $x10^9$)	4 (4.1)	36 (36.7)
Low (21-50 $x10^9$)	34 (34.7)	34 (34.7)
Intermediate (51-100 $x10^9$)	9 (9.2)	9 (9.2)
High (>100 $x10^9$)	3 (3.1)	4 (4.1)
No threshold	47 (48)	16 (16.3)
Antiplatelet use in week before transfusion	6 (6.1)	
**Plasma transfusion**		
N. events	*75*	
N. of transfusion events per patient	1 (1 – 2)	
Total units transfused per patient	20	
N. plasma units per event	2 (2 – 4)	
Product ordered by		
Intensivist	59 (78.7)	
Specialist, non-intensivist	3 (4.0)	
Resident	7 (9.3)	
Other	6 (8.0)	
Primary medical specialty of transfusion requestor		
Anesthesiology	37 (49.3)	
Intensivist	10 (13.3)	
Cardiology	4 (5.3)	
Internal medicine	16 (21.3)	
Pulmonology	1 (1.3)	
Surgery	7 (9.3)	
Other	0 (0)	
Lab values		

INR measured before transfusion	71 (94.7)	
INR target	1.4 (1.2 – 1.5)	
N. INR target stated (%)	49 (65.3)	
INR before transfusion	1.7 (1.4 – 3.1)	
INR after transfusion	1.6 (1.3 – 2.4)	
Transfusion policy (%)	*Stated target*	*Used target*
INR >3.0	0 (0)	18 (24)
INR 1.5 – 3.0	11 (14.7)	27 (36)
INR <1.5	38 (50.7)	23 (30.7)
No threshold	26 (34.7)	7 (9.3)
Anticoagulant use in week before transfusion	49 (65.3)	

Data are presented as mean ± standard deviation (SD), median [25th–75th percentile], or count (%).

Abbreviations: *Hb – Hemoglobin; ICU – Intensive Care Unit; INR – International Normalized Ratio; MTP – Massive Transfusion Protocol; RBC – Red Blood Cells; SOFA – Sequential Organ Failure Assessment.*

Supplement table 3. Detailed clinical characteristics of septic shock cohort during ICU stay, stratified by transfusion status.

	**Septic Shock participants**	**Non-transfused**	**Transfused**	***p*-value**
**Red blood cell transfusion**				
N. patients	395 (100)	283 (72)	112 (28)	
Median days on ICU	4 (2 – 8)	3 (2 – 7)	5 (3 – 9)	**0.01**
Blood loss, mL* (SD)	35 (± 104)	19 (± 71)	74 (± 152)	**0.00**
SOFA* (SD)	7 (± 4)	7 (± 4)	8 (± 4)	**0.00**
Laboratory values during ICU stay				

Hb*, g/dL (SD)	10 (± 2.1)	10.7 (± 1.9)	8.5 (± 1.5)	**0.00**
Nadir Hb (SD)	9 (± 2.2)	9.9 (± 1.9)	7.1 (± 1.6)	**0.00**
Anemia [a]	375 (93.4)	265 (91.4)	110 (97.9)	0.06
Mean platelet count *	187 (± 115)	197 (± 115)	167 (± 112)	**0.04**
Mean nadir platelet count	144 (± 104)	157 (± 105)	115 (± 97)	**0.00**
Overall concomitant transfusions during ICU stay				
Received Platelet transfusion	36 (9.1)	-	25 (22.3)	
Received Plasma transfusion	47 (11.9)	-	36 (32.1)	
Received coagulation product	34 (8.6)	-	22 (19.6)	
**Platelet transfusion**				
N. patients	395 (100)	359 (91)	36 (9)	
Median days on ICU	4 (2 – 8)	4 (2 – 7)	5 (3 – 10)	0.07
Blood loss, mL*	35 (± 104)	28 (± 81)	108 (± 219)	**0.00**
SOFA*	7 (± 4)	7 (± 4)	11 (± 5)	**0.00**
Laboratory results during ICU stay				
Mean Platelet count, $10^3/\mu L$* (SD)	187 (± 115)	200 (± 112)	77 (± 77)	**0.00**
Median nadir platelet count	144 (± 104)	156 (± 102)	39 (± 53)	**0.00**
Thrombocytopenia (%) [b]	283 (60)	248 (55.8)	35 (9)	
51 – 150 $x10^3/\mu L$	118 (29.9)	113 (31.5)	5 (13.9)	
21 – 50 $x10^3/\mu L$	33 (8.4)	22 (6.1)	11 (30.6)	
≤20 $x10^3/\mu L$	17 (4.3)	5 (1.4)	12 (33.3)	

Overall concomitant transfusions				
Received RBC transfusion	112 (28.4)		25 (69.4)	
Received Plasma transfusion	47 (11.9)		20 (55.6)	
Received coagulation product	34 (8.6)		15 (41.7)	
**Plasma transfusion**				
N. patients	395 (100)	348 (88)	47 (12)	
Median days on ICU	4 (2 – 8)	4 (2 – 7)	6 (3 -12)	**0.03**
Blood loss, mL* (SD)	35 (± 104)	26 (± 78)	100 (± 203)	**0.00**
SOFA*	7 (± 4)	7 (± 3)	11 (± 5)	**0.00**
Overall concomitant transfusions				
Received RBC transfusion	112 (28.4)		36 (76.6)	
Received Platelet transfusion	36 (9.1)		20 (42.6)	
Received coagulation product	34 (8.6)		20 (42.6)	
Admission INR elevated >1.5				

Data are presented as mean ± standard deviation (SD), median [25th–75th percentile], or count (%). Group differences were evaluated using Student's t-test, Mann–Whitney U test,
Chi-square test, ANOVA, or Kruskal–Wallis test with Bonferroni correction, with statistical significance defined as ***p < 0.05****. [a] Anemia defined as hemoglobin <12 g/dL for women and <13 g/dL for men (World Health Organization). [b] Thrombocytopenia defined as platelet count <150 cells ×10^9/L, during ICU stay. * Weighted by ICU length of stay (days).*

Abbreviations: *APTT – Activated Partial Thromboplastin Time; Hb – Hemoglobin; ICU – Intensive Care Unit; INR – International Normalized Ratio; PT – Prothrombin Time; RBC – Red Blood Cells; SOFA – Sequential Organ Failure Assessment.*

Missing values Table 1-3

Table 1. Baseline Characteristics of the Study Population, and Stratified per Transfusion Status

	**All participants** (N= 799)	**Non-transfused** (N= 482)	**Transfused** (N= 317)	***p*-value**	**Difference *** (95% CI)**	**Missing (N, %)**
**Patient characteristics**						
Age	65 (55 – 75)	65 (56 – 75)	64 (55 – 75)	0.60	1 (-2 – 3)	0 (0)
Female	308 (39)	187 (39)	121 (38)	0.92	1 (-8 – 6)	0 (0)
Medical history **						
Acute coronary syndrome	71 (9)	43 (9)	28 (9)	1.0	0 (-4 – 4)	0 (0)
Chronic kidney disease	109 (14)	59 (12)	50 (16)	0.2	3.5 (-1 – 9)	
Chronic obstructive pulmonary disease	108 (14)	75 (16)	33 (10)	0.58	5 (-1 – 10)	0 (0)
Heart failure	128 (16)	77 (16)	51 (16)	1.0	0 (-5 – 5)	0 (0)
Hematological disease - Benign	11 (1)	7 (3)	4 (1)	1.0	0 (-2 – 1)	0 (0)
Hematological disease - Malign	39 (5)	14 (3)	25 (8)	**0.03**	5 (2 – 8)	0 (0)
Liver failure	39 (5)	15 (3)	24 (8)	0.00	5 (1 – 8)	0 (0)
Other	382 (48)	154 (32)	84 (27)	0.1	6 (-1 – 12)	0 (0)
No comorbidities	222 (28)	135 (28)	87 (27)	0.9	1 (-6 – 7)	0 (0)
**Type of admission**						
Emergency	711 (89)	438 (91)	273 (86)	**0.05**	5 (0 – 9)	0 (0)
Presence of sepsis at admission, without shock	214 (27)	133 (28)	81 (25)	**0.56**	2 (-4 – 8)	0 (0)

Presence of septic shock at admission	395 (49)	255 (53)	140 (44)	**0.02**	9 (2 – 16)	0 (0)
Admitted for other reason (developed sepsis during admission)	190 (24)	94 (20)	96 (30)	**<0.00**	11 (5 – 17)	0 (0)
Surgery <24 h prior to ICU admission	201 (25)	99 (21)	102 (32)	**0.05**	12 (5 – 18)	0 (0)

Please see the next page for the continuation of Table 1.

**Severity scores**						
APACHE IV score at admission	64 (42 – 87)	60 (39 – 83)	72 (47 – 93)	**<0.00**	10 (5 – 14)	29 (3.6)
SOFA score at first 24h of sepsis diagnosis	7 (4 – 10)	7 (4 – 9)	8 (5 – 11)	**<0.00**	2 (1 – 3)	168 (21)
**Department prior to ICU admission**						
Emergency room	293 (37)	196 (41)	97 (31)	**<0.00**	10 (3 – 17)	0 (0)
Hospital ward	249 (31)	136 (28)	113 (36)	**0.03**	7 (1 – 15)	0 (0)
Operating room	157 (20)	84 (17)	73 (23)	0.06	6 (-0 – 11)	0 (0)
Other hospital	98 (12)	65 (14)	33 (10)	0.24	3 (-2 – 8)	0 (0)
Other	2 (0)	1 (0)	1 (0)	1.0	0 (-1 – 1)	0 (0)
**Additional supportive therapy at admission ****						
Mechanical ventilation	380 (48)	219 (45)	161 (51)	0.15	5 (-2 – 12)	0 (0)
Renal replacement therapy	75 (9)	37 (8)	38 (12)	0.05	4 (0 – 9)	0 (0)
ECMO	5 (1)	0 (0)	5 (1)	**0.01**	-	0 (0)
Other support	23 (3)	11 (2)	12 (4)	0.6	2 (-1 – 4)	0 (0)
**Laboratory results (at admission)**						
Hemoglobin (g/dL)	11.4 (± 2.8)	12.3 (± 2.5)	10.0 (± 2.8)	**<0.00**	2.3 (1.8 – 2.7)	115 (14.4)
Platelet count (x10^9/L)	202 (136 – 288)	208 (145 – 287)	189 (108 – 293)	**0.02**	23 (3 – 42)	129 (16.1)

INR	1.2 (1.1 – 1.5)	1.2 (1.1 – 1.4)	1.4 (1.1 – 1.7)	**<0.00**	0.1 (0 – 0.2)	376 (47.1)
Prothrombin time (sec)	14 (13 – 17)	15 (13 – 17)	14 (13 – 17)	0.9	0 (-2 – 3)	759 (95)
aPTT (sec)	32 (28 – 38)	31 (28 – 36)	33 (28 – 42)	**<0.00**	2 (1 – 4)	378 (47.3)
**Hematological anomalies (at admission)**						
Anemia	460 (58)	238 (49)	222 (70)	**<0.00**	21 (14 – 27)	115 (14.4)
Thrombocytopenia	207 (26)	111 (23)	96 (30)	**0.01**	7 (1 – 14)	129 (16.1)
Elevated INR (>1.5)	97 (12)	38 (8)	59 (19)	**<0.00**	11 (6 – 16)	376 (47.1)
Elevated aPTT (>36 sec)	135 (17)	62 (13)	73 (23)	**<0.00**	10 (5 – 16)	378 (47.3)

Data are expressed as mean ± standard deviation, median [25th–75th percentile], or number (%). Statistical comparisons were performed using the Student's t-test, Mann–Whitney U test, or Chi-square test, with a significance threshold of ***p<0.05****. No Bonferroni correction was applied, results should be interpreted accordingly.* **: multiple options possible. ****Difference: Hodges Lehman median difference, Mean difference and Percentage difference with 95% confidence interval.*

Abbreviations: *APACHE IV – Acute Physiology and Chronic Health Evaluation IV; aPTT – Activated Partial Thromboplastin Time; CI – Confidence interval; ECMO – Extracorporeal Membrane Oxygenation; ICU – Intensive Care Unit; INR – International Normalized Ratio; RBC – Red Blood Cells; SOFA – Sequential Organ Failure Assessment.*

Table 2. Clinical Characteristics of Blood Transfusion Events

**RBC transfusions**		**Missing (N, %)**
N. events	656	0 (0)
N. of transfusion events per patient	2 (1 – 3)	0 (0)
Total units transfused per patient	2 (1 – 5)	0 (0)
N. RBC units per event	1 (1 – 2)	0 (0)
Product ordered by		0 (0)
Intensivist	464 (71)	-
Specialist, nonintensivist	22 (3)	-
Resident	94 (14)	-
Other	76 (12)	-
**Primary medical specialty of transfusion requestor**		1 (0.2)
Anesthesiology	316 (48)	-
Intensivist	149 (24)	-

Cardiology	9 (1)		-
Internal medicine	116 (18)		-
Pulmonology	32 (5)		-
Surgery	29 (4)		-
Other	4 (1)		-
**Hemoglobin values**			
Hb measured prior to transfusion event	629 (96)		15 (2.3)
Hb before transfusion (g/dL)	7.5 (± 1.4)		45 (6.9)
Hb post transfusion (g/dL)	8.9 (± 1.3)		131 (20)
Hb increase after transfusion (g/dL)	1.4 (± 1.3)		131 (20)
Pre-defined threshold (g/dL)	8 (7 – 9)		213 (32.5)
**Transfusion policy**	**Stated target**	**Used target**	
Restrictive (<7 g/dL)	149 (23)	232 (35)	0 (0)
Intermediate (7–9 g/dL)	157 (24)	323 (49)	0 (0)
Liberal (>9 g/dL)	137 (21)	56 (9)	0 (0)
No threshold	213 (33)	45 (7)	0 (0)
N. MTP	13 (2)		0 (0)
N. Whole blood	6 (1)		0 (0)

Please see the next page for the continuation of Table 2.

**Platelet transfusion**		
N. events	203	

N. of transfusion events per patient	1 (1 – 2)		0 (0)
Total units transfused per patient	2 (1 – 6)		0 (0)
N. platelet units per event	1 (1 – 2)		0 (0)
**Product ordered by**			0 (0)
Intensivist	149 (73)		-
Specialist, nonintensivist	34 (17)		-
Resident	17 (8)		-
Other	3 (2)		-
**Primary medical specialty of transfusion requestor**			2 (1)
Anesthesiology	64 (32)		-
Intensivist	44 (22)		-
Cardiology	2 (1)		-
Internal medicine	78 (39)		-
Pulmonology	2 (1)		-
Surgery	8 (4)		-
Other	3 (2)		-
**Platelet values**			
Platelets measured prior to transfusion event	184 (91)		19 (9.4)
Platelet count before transfusion (x10^9 cells/L)	26 (16 – 51)		19 (9.4)
Platelet count post transfusion (x10^9 cells/L)	54 (27 – 78)		78 (38.4)
Platelet count increase (x10^9 cells/L)	15 (4 – 32)		78 (38.4)
Transfusion policy	*Stated target*	*Used target*	
Minimal (≤20 x10^9 cells/L)	21 (10)	65 (32)	0 (0)
Low (21-50 x10^9 cells/L)	62 (31)	71 (35)	0 (0)

Intermediate (51-100 $x10^9$ cells/L)	12 (6)	37 (18)	0 (0)
High (>100 $x10^9$ cells/L)	12 (6)	10 (5)	0 (0)
No threshold	96 (47)	19 (9)	0 (0)
Antiplatelet use in prior week	12 (6)		0 (0)

Please see the next page for the continuation of Table 2.

**Plasma transfusion**		
N. events	180	
N. of transfusion events per patient	1 (1 – 2)	0 (0)
Total units transfused per patient	3 (2 – 5)	0 (0)
N. plasma units per event	2 (1 – 3)	0 (0)
**Product ordered by**		0 (0)
Intensivist	136 (76)	-
Specialist, nonintensivist	30 (17)	-
Resident	14 (8)	-
Other	0 (0)	-
**Primary medical specialty of transfusion requestor**		0 (0)
Anesthesiology	104 (58)	-
Intensivist	25 (14)	-
Cardiology	5 (3)	-
Internal medicine	26 (14)	-
Pulmonology	9 (5)	-
Surgery	10 (6)	-
Other	1 (1)	-
**Lab values**		
INR measured before transfusion	152 (84)	28 (15.6)
INR target	1.3 (1.2 – 1.5)	73 (40.6)
N. INR target stated	107 (59)	73 (40.6)
INR before transfusion	1.6 (1.3 – 2.4)	28 (15.6)

INR after transfusion	1.5 (1.2 – 2)		56 (31.1)
INR decrease after transfusion	0.2 (0.6 – 0.0)		56 (31.1)
**Transfusion policy**	**Stated target**	**Used target**	
INR >3.0	0 (0)	26 (14)	0 (0)
INR 1.5 – 3.0	21 (12)	56 (31)	0 (0)
INR <1.5	86 (48)	70 (39)	0 (0)
No threshold	73 (41)	28 (16)	0 (0)
Anticoagulant use in week before transfusion	75 (42)		0 (0)

Data are presented as mean ± standard deviation (SD), median [25th–75th percentile], or count (%).

Abbreviations: *Hb – Hemoglobin; ICU – Intensive Care Unit; INR – International Normalized Ratio; MTP – Massive Transfusion Protocol; RBC – Red Blood Cells; SOFA – Sequential Organ Failure Assessment.*

Table 3. Detailed Clinical Characteristics During Intensive Care Unit Stay, Stratified by Transfusion Status.

	**All participants**	**Non- transfused**	**Transfused**	***p*-value**	**Difference (95% CI)****	**Missing (N, %)**
**Red blood cell transfusion**						
N. patients	799 (100)	530 (76)	269 (34)			
Median ICU stay, days	5 (3 – 11)	5 (3 – 9)	8 (4 – 16)	**<0.00**	3 (2 – 3)	0 (0)
Blood loss, mL*	40 (± 120)	15 (± 58)	88 (± 182)	**<0.00**	72 (49 – 94)	1 (0.1)
SOFA*	6.6 (± 4)	6.2 (± 4)	7.6 (± 4)	**<0.00**	1.4 (0.8 – 2.1)	102 (12.8)
**Laboratory values during ICU stay**						
Hb*	10 (± 2.0)	10.8 (± 1.9)	8.6 (± 1.3)	**<0.00**	2.3 (2.0 – 2.5)	226 (28.3)
Nadir Hb	8.9 (± 2.2)	10 (± 1.9)	7.5 (± 1.4)	**<0.00**	2.7 (2.4 – 3.0)	226 (28.3)
Anemia at admission	460 (58)	266 (50)	194 (72)	**<0.00**	22 (15 – 29)	115 (14.4)
Anemia [a]	744 (93)	478 (90)	266 (99)	**<0.00**	9 (6 –12)	
**Platelet transfusion**						

N. patients	799 (100)	721 (90)	78 (10)			
Median ICU stay, days	5 (3 – 11)	5 (3 – 10)	8 (4 – 18)	**<0.00**	2 (1 – 3)	0 (0)
Blood loss, mL *	40 (± 120)	32 (± 106)	110 (± 197)	**<0.00**	78 (33 – 123)	1 (0.1)
SOFA *	6.6 (± 3.9)	6.3 (± 3.7)	10.1 (± 4.5)	**<0.00**	3.8 (2.7 – 5)	102 (12.8)
**Laboratory results during ICU stay**						
Platelet count *	197 (± 120)	210 (± 117)	77 (± 77)	**<0.00**	133 (109 – 157)	273 (34.2)
Nadir platelet count	126 [68 – 195]	138 [86 –203]	23 [10 – 42]	**<0.00**	120 (105 – 136)	273 (34.2)
Admission thrombocytopenia	207 (26)	160 (22)	47 (60)	**<0.00**	38 (27 – 49)	129 (16.1)
Thrombocytopenia [b]	453 (57)	378 (52)	75 (96)	**<0.00**	44 (38 – 49)	273 (34.2)
51 – 150 $x10^9$ cells/L	326 (41)	306 (42)	20 (26)	**<0.00**	17 (6 – 27)	**-**
21 – 50 $x10^9$ cells/L	85 (11)	57 (8)	28 (36)	**<0.00**	28 (17 –39)	**-**
≤20 $x10^9$ cells/L	42 (5)	15 (2)	27 (35)	**<0.00**	33 (22 – 43)	-

Please see the next page for the continuation of Table 2

**Plasma transfusion**						
N. patients	799 (100)	691 (87)	108 (13)			
Median ICU stay, days	5 (3 – 11)	5 (3 – 10)	8 (4 – 16)	**<0.00**	2 (1 – 3)	0 (0)
Blood loss, mL*	40 (120)	24 (78)	139 (240)	**<0.00**	115 (69 – 161)	1 (0.1)
SOFA*	6.6 (± 3.9)	6.2 (± 3.6)	9.3 (± 4.7)	**<0.00**	3 (2 – 4)	102 (12.8)
Admission INR elevated >1.5	97 (12)	72 (10)	25 (23)	**0.02**	13 (5 –21)	376 (47.1)

Data are presented as mean ± standard deviation (SD), median [25th–75th percentile], or count (%). Group differences were evaluated using Student's t-test, Mann– Whitney U test, Chi-square test, ANOVA, or Kruskal–Wallis test with Bonferroni correction, with statistical significance defined as ***p < 0.05****. [a] Anemia defined as hemoglobin <12 g/dL for women and <13 g/dL for men (World Health Organization). [b] Thrombocytopenia defined as platelet count <150 cells ×10^9/L, during ICU stay. * Weighted by ICU length of stay (days). **Difference: Hodges Lehman median difference, Mean difference and Percentage difference with 95% confidence interval.*

Abbreviations: *APTT – Activated Partial Thromboplastin Time; CI – Confidence Interval; Hb – Hemoglobin; ICU – Intensive Care Unit; INR – International Normalized Ratio; RBC – Red Blood Cells; SOFA – Sequential Organ Failure Assessment.*
